# Reward and punishment in climate change dilemmas

**DOI:** 10.1038/s41598-019-52524-8

**Published:** 2019-11-07

**Authors:** António R. Góis, Fernando P. Santos, Jorge M. Pacheco, Francisco C. Santos

**Affiliations:** 1 INESC-ID and Instituto Superior Técnico, Universidade de Lisboa, IST-Taguspark, 2744-016 Porto, Salvo Portugal; 2 ATP-group, P-2744-016 Porto, Salvo Portugal; 3 Unbabel, R. Visc. de Santarém 67B, 1000-286 Lisboa, Portugal; 4 Department of Ecology and Evolutionary Biology, Princeton University, Princeton, USA; 5 Centro de Biologia Molecular e Ambiental, Universidade do Minho, 4710 - 057 Braga, Portugal; 6 Departamento de Matemática e Aplicações, Universidade do Minho, 4710 - 057 Braga, Portugal; 7 Machine Learning Group, Université Libre de Bruxelles, Boulevard du Triomphe CP212, 1050, Bruxelles, Belgium

**Keywords:** Nonlinear phenomena, Computational science

## Abstract

Mitigating climate change effects involves strategic decisions by individuals that may choose to limit their emissions at a cost. Everyone shares the ensuing benefits and thereby individuals can free ride on the effort of others, which may lead to the tragedy of the commons. For this reason, climate action can be conveniently formulated in terms of Public Goods Dilemmas often assuming that a minimum collective effort is required to ensure any benefit, and that decision-making may be contingent on the risk associated with future losses. Here we investigate the impact of reward and punishment in this type of collective endeavors — coined as collective-risk dilemmas — by means of a dynamic, evolutionary approach. We show that rewards (positive incentives) are essential to initiate cooperation, mostly when the perception of risk is low. On the other hand, we find that sanctions (negative incentives) are instrumental to maintain cooperation. Altogether, our results are gratifying, given the a-priori limitations of effectively implementing sanctions in international agreements. Finally, we show that whenever collective action is most challenging to succeed, the best results are obtained when both rewards and sanctions are synergistically combined into a single policy.

## Introduction

Climate change stands as one of our biggest challenges in what concerns the emergence and sustainability of cooperation^1,2^. Indeed, world citizens build up high expectations every time a new International Environmental Summit is settled, unfortunately with few resulting solutions implemented so far. This calls for the development of more effective incentives, agreements and binding mechanisms. The problem can be conveniently framed resorting to the mathematics of game theory, being a paradigmatic example of a Public Goods Game^3^: at stake there is a global good from which every single individual can profit, irrespectively of contributing to maintain it. Parties may free ride on the efforts of others, avoiding any effort themselves, while driving the population into the tragedy of the commons^4^. Moreover, since here cooperation aims at averting collective losses, this type of dilemmas is often referred as public *bad* games, in which achieving collective goals often depends on reaching a threshold number of cooperative group members^5–8^.

One of the multiple obstacles attributed to such agreements is misperceiving the actual risk of future losses, which significantly affects the ensuing dynamics of cooperation^5,9^. Another problem relates to both the incapacity to sanction those who do not contribute to the welfare of the planet, and/or to reward those who subscribe to green policies^10^. Previous cooperation studies show that reward (positive incentives), punishment (negative incentives) and the combination of both^11–23^ have a different impact depending on the dilemma in place. Assessing the impact of reward and punishment (isolated or combined) in the context of N-person threshold games — and in the particular case of climate change dilemmas — remains, however, an open problem.

Here we study, theoretically, the role of both institutional reward and punishment in the context of climate change agreements. Previous works consider the public good as a linear function of the number of contributors^12,17,21,22^ and conclude that punishment is more effective than reward (for an optimal combination of punishment and reward see ref.^12^). We depart from this linear regime by modeling the returns on the public good as a threshold problem, combined with an uncertain outcome, represented by a risk of failure. As a result – and as detailed below – the dynamical portrait of our model reveals new internal equilibria^9^, allowing to identify the dynamics of coordination and coexistence typifying collective action problems. As discussed below, the reward and punishment mechanisms will impact, in a non-trivial way, those equilibria.

We consider a population of size *Z*, where each individual can be either a Cooperator (***C***) or a Defector (***D***), when participating in a N-player Collective-Risk dilemma (**CRD**)^5,9,10,24–30^. In this game, each participant starts with an initial endowment *B* (viewed as the asset value at stake) that may be used to contribute to the mitigation of the effects of climate change. A cooperator incurs a cost corresponding to a fraction *c* of her initial endowment *B*, in order to help prevent a collective failure. On the other hand, a defector refuses to have any cost, hoping to free ride on the contributions of others. We require a minimum number of 0 < *M* ≤ *N* cooperators in a group of size *N* before collective action is realized; if a group of size $N$ does not contain at least *M C*s, all members lose their remaining endowments with a probability *r*, where *r* (0 ≤ *r* ≤ 1) stands as the risk of collective failure. Otherwise, everyone will keep whatever she has. This **CRD** formulation has been shown to capture some of the key features discovered in recent experiments^5,24,31–33^, while highlighting the importance of risk. In addition, it allows one to test model parameters in a systematic way that is not possible in human experiments. Moreover, the adoption of non-linear returns mimics situations common to many human and non-human endeavors^6,34–41^, where a minimum joint effort is required to achieve a collective goal. Thus, the applicability of this framework extends well beyond environmental governance, given the ubiquity of such type of social dilemmas in nature and societies.

Following Chen *et al*.^12^, we include both reward and punishment mechanisms in this model. A fixed group budget *Nδ* (where *δ* ≥ 0 stands for a per-capita incentive) is assumed to be available, of which a fraction *w* is applied to a reward policy and the remaining 1-*w* to a punishment policy. We assume the effective impact of both policies to be equivalent, meaning that each unit spent will directly increase/decrease the payoff of a cooperator/defector by the same amount. For details on policies with different efficiencies, see Methods.

Instead of considering a collection of rational agents engaging in one-shot Public Goods Games^32,42^, here we adopt an evolutionary description of the behavioral dynamics^9^, in which individuals tend to copy those appearing to be more successful. Success (or fitness) of individuals is here associated with their average payoff. All individuals are equally likely to interact with each other, causing all cooperators and defectors to be equivalent, on average, and only distinguishable by the strategy they adopt. Therefore, and considering that only two strategies are available, the number of cooperators is sufficient to describe any configuration of the population. The number of individuals adopting a given strategy (either ***C*** or ***D***) evolves in time according to a stochastic birth–death process^43,44^, which describes the time evolution of the social learning dynamics (with exploration): At each time-step each individual (*X*, with fitness *f*_X_) is given the opportunity to change strategy; with probability *μ*, *X* randomly explores the strategy space^45^ (a process similar to mutations in a biological context that precludes the existence of absorbing states). With probability (1-*μ*), *X* may adopt the strategy of a randomly selected individual (*Y*, with fitness *f*_Y_), with a probability that increases with the fitness difference (*f*_Y_*–f*_X_)^44^. This renders the stationary distribution (see Methods) an extremely useful tool to rank the most visited states given the ensuing evolutionary dynamics of the population. Indeed, the stationary distribution provides the prevalence of each of the population’s possible configuration, in terms of the number of ***C***s (*k*) and ***D***s (*Z-k)*. Combined with the probability of success characterizing each configuration, the stationary distribution can be used to compute the overall success probability of a given population – the average group achievement, *η*_*G*_. This value represents the average fraction of groups that will overcome the **CRD**, successfully preserving the public good.

## Results

**Figure 1:**
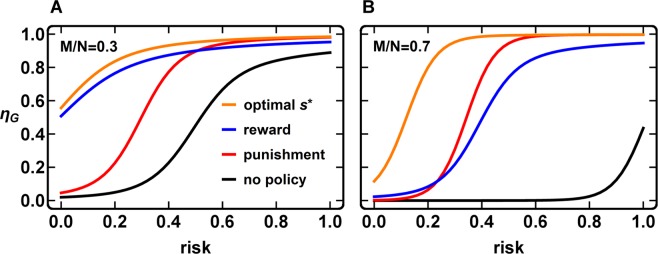
Average group achievement *η*_*G*_ as a function of risk. Left: Group relative threshold M/N = 3/10. Right: Group relative threshold M/N = 7/10. In both panels, the black line corresponds to a reference scenario where no policy is applied. The red line shows *η*_*G*_ in the case where all available budget is applied to pure-Punishment (*w* = 0), whereas the blue line shows results for pure-Reward (*w* = 1). Pure-Reward is most effective at low risk values, while pure-Punishment is marginally the most effective policy at high risk. These features are more pronounced for low relative thresholds (left panel), and only at high thresholds does pure-Punishment lead to a sizeable improvement with respect to pure-Reward. Finally, the orange line shows the results using the combination of Reward and Punishment, leading (naturally) to the best results. In this case, we adopt pure-Reward (*w* = 1) when there are few cooperators and, above a certain critical point *k*/*Z* = *s* = 0.5, we switch to pure-Punishment (*w* = 0). As detailed in the main text (see Fig. 3 and Methods), *s* = 0.5 provides the optimal switching point s* for cooperation to thrive. Other parameters: Population size Z = 50, group size *N* = 10, cost of cooperation *c* = 0.1, initial endowment *B = *1, budget *δ* = 0.025, reward efficiency *a* = 1, punishment efficiency *b* = 1, intensity of selection *β* = 5, mutation rate *µ* = 0.01.

In Fig. 1 we compare the average group achievement *η*_*G*_ (as a function of risk) in four scenarios: (*i)* a reference scenario without any policy (*i*.*e*., no reward or punishment, in black); and three scenarios where a budget is applied to (*ii)* rewards, (*iii)* punishment and (*iv)* a combination of rewards and sanctions (see below). Our results are shown for the two most paradigmatic regimes: low (Fig. 1A) and high (Fig. 1B) coordination requirements. Naturally *η*_*G*_ improves whenever a policy is applied. Less obvious is the difference between the various policies. Applying only rewards (blue curves in Fig. 1) is more effective than only punishment (red curve) for low values of risk. The opposite happens when risk is high. On scenarios with a low relative threshold (Fig. 1A), rewards play the key role, with sanctions only marginally outperforming them for very high values of risk. For high coordination thresholds (Fig. 1B) reward and punishment portray comparable efficiency in the promotion of cooperation, with pure-Punishment (*w* = 0) performing slightly better than pure-Reward (*w* = 1).

**Figure 2:**
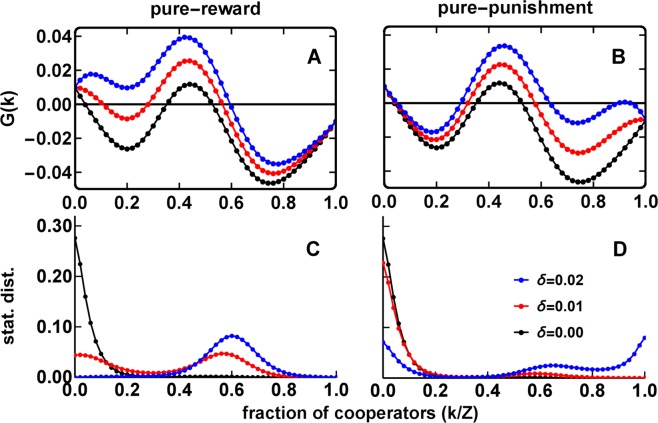
Gradient of selection (top panels, A and B) and stationary distribution (bottom panels, C and D) for the different values of per-capita budget *δ* indicated, using either pure-Reward (*w* = 1, left panels) or pure-Punishment (*w* = 0, right panels). The black curve is equal on the left and right panels, since in this case *δ* = 0. As *δ* increases, the behaviour under Reward and Punishment is qualitatively similar, by displacing the (unstable) coordination equilibrium towards lower values of *k/Z*, while displacing the (stable) coexistence equilibrium towards higher values of *k/Z*. This happens, however, only for low values of *δ*. Indeed, by further increasing *δ* one observes very different behaviours under Reward and Punishment: Whereas under Punishment the equilibria are further moved apart (in accord with what happened for low *δ*) under Reward the coordination equilibrium disappears, and the overall dynamics becomes characterized by a single coexistence equilibrium which consistently shifts towards higher values of *k*/*Z* with increasing *δ*. This difference in behaviour, in turn, has a dramatic impact in the overall prevalence of configurations achieved by the population dynamics, as shown by the stationary distributions: On panel C (pure-Reward) the population spends most of the time on intermediate states of cooperation. On panel D (pure-Punishment) the population spends most of the time on both extremes (high and low cooperation) but especially on low cooperation states. Other parameters: *Z* = 50, *N* = 10, *M* = 5, *c* = 0.1, *B* = 1, *r* = 0.5, *a* = *b* = 1, *β* = 5 and µ = 0.01 (see Methods for details).

Justifying these differences is difficult from the analysis of *η*_*G*_ alone. To better understand the behavior dynamics under Reward and Punishment, we show in Fig. 2 the gradients of selection (top panels) and stationary distributions (lower panels) for each case and different budget values. Each gradient of selection represents, for each discrete state *k*/*Z* (*i*.*e*., fraction of ***C****s*), the difference $G(k)={T}^{+}(k)-{T}^{-}(k)$ among the probability to increase (*T*^+^(*k*)) and decrease (*T*^−^(*k*)) the number of cooperators (see Methods) by one. Whenever *G*(*k*) > 0 the fraction of ***C***s is likely to increase; whenever *G*(*k*) < 0 the opposite is expected to happen. The stationary distributions show how likely it is to find the population in each (discrete) configuration of our system. The panels on the left-hand side show the results obtained for the **CRD** under pure-Reward; on the right-hand side, we show the results obtained for pure-Punishment.

Naturally, both mechanisms are inoperative whenever the per-capita incentives are inexistent (*δ* = 0), creating a natural reference scenario in which to study the impact of Reward and Punishment on the **CRD**. In this case, above a certain value of risk (*r*), decision-making is characterized by two internal equilibria (*i*.*e*., adjacent finite population states with opposite gradient sign, representing the analogue of fixed points in a dynamical system characterizing evolution in infinite populations). Above a certain fraction of cooperators the population overcomes the coordination barrier and naturally self-organizes towards a stable co-existence of cooperators and defectors. Otherwise, the population is condemned to evolve towards a monomorphic population of defectors, leading to the tragedy of the commons^9^. As the budget for incentives increases, using either Reward or Punishment leads to very different outcomes, as depicted in Fig. 2.

Contrary to the case of linear Public Goods Games^12^, in the **CRD** coordination and co-existence dynamics already exist in the absence of any reward/punishment incentive. Reward is particularly effective when cooperation is low (small *k/Z*), showing a significant impact on the location of the finite population analogue of an unstable fixed point. Indeed, increasing *δ* lowers the minimum number of cooperators required to reach the cooperative basin of attraction (as well as increasing the prevalence of cooperators in co-existence point on the right), which ultimately disappears for high *δ* (Fig. 2A). This means that a smaller coordination effort is required before the population dynamics start to naturally favor the increase of cooperators. Once this initial barrier is surpassed, the population will naturally tend towards an equilibrium state, which does not improve appreciably under Reward. The opposite happens under Punishment. The location of the coordination point is little affected, yet once this barrier is overcome, the population will evolve towards a more favorable equilibrium (Fig. 2B). Thus, while Reward seems to be particularly effective to bootstrap cooperation towards a more cooperative *basin of attraction*, Punishment seems effective in sustaining high levels of cooperation.

As a consequence, the most frequently observed configurations are very different when using each of the policies. As shown by the stationary distributions (Fig. 2C,D), under Reward the population visits more often states with intermediate values of cooperation (*i*.*e*., where ***C***s and ***D***s co-exist). Intuitively, this happens because the coordination effort is eased by the rewards, causing the population to effectively overcome it and reach the coexistence point (the equilibrium state with an intermediate amount of cooperators) thus spending most of the time near it. On the other hand, Punishment will not ease the coordination effort, and thus the population will spend most of the time in states of low cooperation, failing to overcome this barrier. Notwithstanding, once surpassed, the population will stabilize on higher states of cooperation. This is especially evident for high budgets, as shown with *δ* = 0.*02* (blue line). Moreover, since *Nδ* corresponds to a fixed total amount which is distributed by the existing cooperators/defectors, this causes the per-cooperator/defector budget to vary depending on the number of existing cooperators/defectors (*i*.*e*., each of the *j* cooperators receives *wδN/j* and each defector loses (1 − *w*)*δN/*(*N* *−* *j*)). In other words, positive (negative) incentives become very profitable (or severe) if defection (cooperation) prevails within a group. In particular, whenever the budget is significant (see, *e*.*g*., *δ* = 0.02 in Fig. 2) the punishment becomes so high when there are few defectors within a group, that a new equilibrium emerges close to full cooperation.

The results in Fig. 2 show that Reward can be instrumental in fostering pro-social behavior, while Punishment can be used for its maintenance. This suggests that, to combine both policies synergistically, pure-Reward (*w* = 1) should be applied at first, when there are few cooperators (low *k*/*Z*); above a certain critical point (*k*/*Z* = *s*) one should switch to pure-Punishment (*w* = 0). In the Methods section, we demonstrate that, similar to linear Public Goods Games^12^, in **CRD**s this is indeed the policy which minimizes the advantage of the defector, even if we consider the alternative possibility of applying both policies simultaneously. In Methods, we also compute a general expression for the optimal switching point s*, that is, the value of *k* above which Punishment should be applied instead of Reward to maximize cooperation and group achievement. By using such policy — that we denote by s* — we obtain the best results shown with an orange line in Fig. 1. We propose, however, to explore what happens in the context of a **CRD** when s* is not used. How much cooperation is lost when we deviate from s* to either of the pure policies, or to a policy which uses a switching point different from the optimal one?

**Figure 3:**
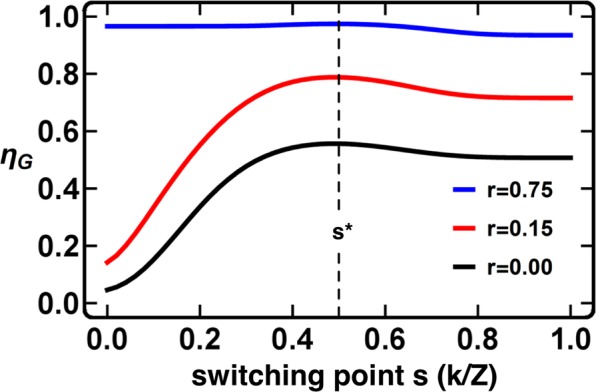
Average group achievement *η*_*G*_ as a function of the location of the switching point *s*. The switching point *s* corresponds to the configuration (fraction of **C**s in the population, *k*/*Z*) above which *w* suddenly switches from pure-Reward (*w* = 1) to pure-Punishment (*w* = 0). Assuming both policies are equally efficient, the optimal switching point occurs at 50% of cooperators (*k*/*Z* = 0.5). The far-left values of *s* correspond to a static policy of always pure-Punishment – the switch from pure-Reward to pure-Punishment occurs immediately at 0% of cooperators. On the far-right (switching point = 100%) a pure-Reward policy is depicted. We can also see what happens when the switch occurs too late or too early, for different values of risk. For low values of risk, it is significantly less harmful to have a late switch from Reward to Punishment than an early one, meaning that when the population configuration cannot be precisely observed, it is preferable to keep rewarding for longer. See Methods for the calculation of the optimal switching point (s*) that maximizes cooperation fitness relative to defection – and consequent group achievement. Other parameters: *Z* = 50, *B* = 1, µ = 0.01, *β* = 5, *N* = 10, *M* = 3, *c* = 0.1, and *δ* = 0.025.

Figure 3 illustrates how the choice of the switching point *s* impacts the overall cooperation, as evaluated by *η*_*G*_, for different values of risk. For a switching point of *s* = *k*/*Z* = 1.0 (0.0) a static policy of always pure-Reward (pure-Punishment) is used. This can be seen on the far right (left) of Fig. 3. Figure 3 suggests that, for low thresholds, an optimal policy switching (which, for the parameters shown, occurs for *s* = 50%, see Methods) is only marginally better than a policy solely based on rewards (*s* = 1). Figure 3 also allows for a comparison of what happens when the switching point occurs too late (excessive rewards) or too early (excessive sanctions) in a low-threshold scenario. A late switch is significantly less harmful than an early one. In other words, our results suggest that when the population configuration cannot be precisely observed, it is preferable to keep rewarding for longer. This said, whenever the perception of risk is high (an unlikely situation these days) an early switch is slightly less harmful than a late one. In the most difficult scenarios, where stringent coordination requirements (large *M*) are combined with a low perception of risk (low *r*), the adoption of a combined policy becomes necessary (see right panel of Fig. 1).

## Discussion

One might expect the impact of Reward and Punishment to lead to symmetric outcomes – Punishment would be effective for high-cooperation the same way that Reward is effective for low-cooperation. In low-cooperation scenarios (under low risk, threshold or budget) Reward alone plays the most important role. However, in the opposite scenario, Punishment alone does not have the same impact. Either a favourable scenario occurs, where any policy yields a satisfying result, or Punishment cannot improve outcomes on its own. In the latter case, the synergy between both policies becomes essential to achieve cooperation. Such optimal policy involves a combination of the single policies, Reward and Punishment, which is dynamic, in the sense that the combination does not remain the same for all configurations of the population. It corresponds to employing pure Reward at first, when cooperation is low, switching subsequently to Punishment whenever a pre-determined level of cooperation is reached.

The optimal procedure, however, is unlikely to be realistic in the context of Climate Change agreements. Indeed, and unlike other Public Goods Dilemmas, where Reward and Punishment constitute the main policies available for Institutions to foster cooperative collective action, in International Agreements it is widely recognized that Punishment is very difficult to implement^2,42^. This has been, in fact, one of the main criticisms put forward in connection with Global Agreements on Climate Mitigation: They suffer from the lack of sanctioning mechanisms as it is practically impossible to enforce any type of sanctioning at a Global level. In this sense, the results obtained here by means of our dynamical, evolutionary approach, are gratifying, given these a-priori limitations of sanctioning in **CRD**s. Not only do we show that Reward is essential to foster cooperation, mostly when both the perception of risk is low and the overall number of engaged parties is small (low *k*/*Z*), but also we show that Punishment mostly acts to sustain cooperation, after it has been installed. Given that low-risk scenarios are more common and harmful to cooperation than high-risk ones, our results in connection with rewards provide a viable way to explore in the quest for establishing Global cooperative collective action. Reward policies may also be very relevant in scenarios where Climate Agreements are coupled with other International agreements from which parties are not interested to deviate from^2,42^. Finally, the fact that rewards ease coordination towards cooperative states suggests that positive incentives should also be used within intervention mechanisms aiming at fostering pro-sociality in artificial systems and hybrid populations comprising humans and machines^46–49^.

The model used takes for granted the existence of an institution with a budget available to implement either Reward or Punishment. New behaviours may emerge once individuals are called to decide whether or not to contribute to such an institution, allowing for a scenario where this institution fails to exist^10,28,50,51^. At present, and under the Paris agreement, we are witnessing the potential birth of an informal funding institution, whose goal is to finance developing countries to help them increase their mitigation capacity. Clearly, this is just an example pointing out to the fact that the prevalence of local and global institutional incentives may depend and may be influenced by the distribution of wealth available among parties, in the same way that it influences the actual contributions to the public good^10,29,33^. Finally, several other effects may further influence and/or affect the present results. Among others, if intermediate tasks are considered^33^, or if individuals have the opportunity to pledge their contribution before their actual action^7,40,52^, it is likely that pro-social behavior may be enhanced. Work along these lines is in progress.

## Methods

### Public goods and collective risks

Let us consider a population with *Z* individuals, where each individual can be a cooperator (***C***) or a defector (***D***). For each round of this game, a group of *N* players is sampled from the original finite population of size *Z*, which corresponds to a process of sampling without replacement. The probability of a group comprising any possible combination of ***C***s and ***D***s is given by the hypergeometric distribution. In the context of a given group, a strategy is associated with a payoff value corresponding to an individual’s earnings in that round, which depend on the action of the rest of group. Fitness is the expected payoff of an individual in a population, before knowing to which group he was assigned. This way, for a population with *k* out of *Z C*s and each group containing *j* out of *N C*s, the fitness of a *D* and a *C* can be written as:

1 $$\begin{array}{c}{f}_{D}={(\begin{array}{c}Z-1\\ N-1\end{array})}^{-1}\mathop{\sum }\limits_{j=0}^{N-1}(\begin{array}{c}k\\ j\end{array})(\begin{array}{c}Z-k-1\\ N-j-1\end{array}){\Pi }_{{\rm{D}}}(j)\end{array}$$

2 $$\begin{array}{c}{f}_{C}={(\begin{array}{c}Z-1\\ N-1\end{array})}^{-1}\mathop{\sum }\limits_{j=0}^{N-1}(\begin{array}{c}k-1\\ j\end{array})(\begin{array}{c}Z-k\\ N-j-1\end{array}){\Pi }_{{\rm{C}}}(j+1)\end{array}$$

where ${\Pi }_{{\rm{C}}}(j)$ and ${\Pi }_{{\rm{D}}}(j)$ stand for the payoff or a *C* and a *D* in a single round, in a group with N players and *j Cs*. To define the payoff functions, let $\theta (x)$ be a Heaviside step-function distribution, where *θ*(*x*) = 0 if *x* < 0 and *θ*(*x*) = 1 if *x* ≥ 0. Each player can contribute with a fraction *c* of her endowment *B* (with 0 ≤ *c* ≤ 1), and in case a group contains less than *M* cooperators (0 < *M* ≤ *N*) there is a risk *r* of failure (0 ≤ *r* ≤ 1), in which case no player obtains her remaining endowment. The payoff of a defector (${\Pi }_{D}(j)$) and the payoff of a cooperator (${\Pi }_{C}(j)$), before incorporating any policy, can be written as^9^:

3 $$\begin{array}{c}{\Pi }_{D}(j)=B\{\theta (j-M)+(1-r)[1-\theta (j-M)]\}\end{array}$$

4 $$\begin{array}{c}{\Pi }_{C}(j)={\Pi }_{D}(j)-cB\end{array}$$

### Reward and punishment

To include a Reward or a Punishment policy, let us follow ref.^12^ and consider a group budget *N*∙*δ* which can be used to implement any type of policy. The fraction of *N*∙*δ* applied to Reward is represented by the weight *w*, with *0* ≤ *w* ≤ 1. Parameters *a* and *b* correspond to the efficiency of Reward and Punishment (for all Figures above it was assumed that *a* = *b* = 1).

5 $$\begin{array}{c}{\Pi }_{D}^{P}(j)={\Pi }_{D}(j)-\frac{b(1-w)N\delta }{N-j}\end{array}$$

6 $$\begin{array}{c}{\Pi }_{C}^{R}(j)={\Pi }_{C}(j)+\frac{awN\delta }{j}\end{array}$$

Naturally, these new payoff functions can be included into the previous fitness functions (${\Pi }_{D}^{P}$ replaces ${\Pi }_{D}$ and ${\Pi }_{C}^{R}$ replaces ${\Pi }_{C}$), letting fitness values account for the different policies.

### Evolutionary dynamics in finite populations

The fitness functions written above allow us to setup the (discrete time) evolutionary dynamics. Indeed, the configurations of the entire population may be used to define a Markov Chain, where each state is characterized by number of cooperators^9,44^. To decide in which direction the system will evolve, at each step a player *i* and a neighbour *j* of her are drawn at random from the population. Player *i* decides whether to imitate her neighbour *j* with a probability depending on the difference between their fitness^43,44^. This way, a system with *k* cooperators may stay in the same state, switch to *k* − 1 or to *k* + 1. The probability of player *i* imitating player *j* can be given by the Fermi function:

7 $$\begin{array}{c}{p}_{j,i}(k)\equiv {[1+{e}^{-\beta ({f}_{j}-{f}_{i})}]}^{-1}\end{array}$$

where *β* is the intensity of selection. Using this probability distribution, we can fully characterize this Markov process. Let *k* be the total number of cooperators in the population and *Z* the total size of the population. ${T}^{+}(k)$ and ${T}^{-}(k)$ are the probabilities to increase and decrease *k* by one, respectively^44^:

8 $$\begin{array}{c}{T}^{\pm }(k)=\,\frac{k}{Z}\,\frac{Z-k}{Z}\,{[1+{e}^{\mp \beta [{f}_{C}(k)-{f}_{D}(k)}]}^{-1}\end{array}$$

The most likely direction can be computed using the difference $G(k)\equiv {T}^{+}(k)-{T}^{-}(k)$. A mutation rate can be introduced by using transition probabilities ${T}_{\mu }^{+}(k)=(1-\mu ){T}^{+}(k)+\mu \frac{Z-k}{Z}$ and ${T}_{\mu }^{-}(k)=(1-\mu ){T}^{-}(k)+\mu \frac{k}{Z}$. In all cases we used a mutation rate *μ* = 0.01, this way avoiding the population to fixate in a monomorphic configuration. In this context, the stationary distribution becomes a very useful tool to analyse the overall population dynamics, providing the probability ${\bar{p}}_{k}=P(\frac{k}{Z})$ for each of the *Z* + 1 states of this Markov Chain to be occupied^53,54^. For each given population state *k*, the hypergeometric distribution can be used to compute the average fraction of groups that obtain success −*a*_*G*_(*k*). Using the stationary distribution and the average group success, the average group achievement (*η*_*G*_) can then be computed, providing the overall probability of achieving success: ${\eta }_{G}=\mathop{\sum }\limits_{k=0}^{Z}{\bar{p}}_{k}{a}_{G}(k)$.

### Combined policies

By allowing the weight *w* to depend on the frequency of cooperators, we can derive the optimal switching point s* between positive and negative incentives by minimizing the defector’s advantage (*f*_D_ − *f*_C_). This is done similarly to ref.^12^, but using finite populations and therefore a hypergeometric distribution (see Eqs (1), (2), (5), and (6)), to account for sampling without replacement. From Eqs (1) and (2), we get

$$\begin{array}{rcl}{f}_{D} & = & \mathop{\sum }\limits_{j=0}^{N-1}\frac{(\begin{array}{c}\,k\\ j\end{array})(\begin{array}{c}Z-1-k\\ N-1-j\end{array})}{(\begin{array}{c}Z-1\\ N-1\end{array})}({\Pi }_{D}(j)-\frac{b(1-w)N\delta }{N-j})\\ {f}_{C} & = & \mathop{\sum }\limits_{j=0}^{N-1}\frac{(\begin{array}{c}k-1\\ j\end{array})(\begin{array}{c}Z-1-(k-1)\\ N-1-j\end{array})}{(\begin{array}{c}Z-1\\ N-1\end{array})}({\Pi }_{C}(j+1)-c+\frac{awN\delta }{j+1})\end{array}$$

from which we aim at finding the value of *w* (with respect to *k*) that minimizes *F*′ = *f*_D_ − *f*_C_. Since ${\Pi }_{D}(j)$, ${\Pi }_{C}(j+1)$ and *c* do not depend on *w*, these quantities do not affect the choice of the optimal *w*, leaving us with the problem of minimizing the following expression:

$${F}^{\text{'}}=-\,N\delta \mathop{\sum }\limits_{j=0}^{N-1}\frac{(\begin{array}{c}k\\ j\end{array})(\begin{array}{c}Z-1-k\\ N-1-j\end{array})}{(\begin{array}{c}Z-1\\ N-1\end{array})}(\frac{b(1-w)}{N-j})-N\delta \mathop{\sum }\limits_{j=0}^{N-1}\frac{(\begin{array}{c}k-1\\ j\end{array})(\begin{array}{c}Z-k\\ N-1-j\end{array})}{(\begin{array}{c}Z-1\\ N-1\end{array})}(\frac{aw}{j+1})$$

Since $$(\begin{array}{c}k\\ j\end{array})=(\begin{array}{c}k-1\\ j\end{array})\frac{k}{k-j}\,{\rm{and}}\,(\begin{array}{c}Z-1-k\\ N-1-j\end{array})=(\begin{array}{c}Z-k\\ N-1-j\end{array})\frac{Z-k-(N-1-j)}{Z-k},$$

$$\begin{array}{c}F\text{'}=-\,N\delta \mathop{\sum }\limits_{j=0}^{N-1}\frac{(\begin{array}{c}k-1\\ j\end{array})(\begin{array}{c}Z-k\\ N-1-j\end{array})}{(\begin{array}{c}Z-1\\ N-1\end{array})}(\frac{aw}{j+1}+\frac{b(1-w)}{N-j}\frac{k}{k-j}\frac{Z-k-N+1+j}{Z-k})\\ \,=-\,N\delta \mathop{\sum }\limits_{j=0}^{N-1}\frac{(\begin{array}{c}k-1\\ j\end{array})(\begin{array}{c}Z-k\\ N-1-j\end{array})}{(\begin{array}{c}Z-1\\ N-1\end{array})}(w[\frac{a}{j+1}-\frac{b}{N-j}\frac{k}{k-j}\frac{Z-k-N+1+j}{Z-k}])\\ \,\,\,-N\delta \mathop{\sum }\limits_{j=0}^{N-1}\frac{(\begin{array}{c}k-1\\ j\end{array})(\begin{array}{c}Z-k\\ N-1-j\end{array})}{(\begin{array}{c}Z-1\\ N-1\end{array})}\frac{b}{N-j}\frac{k}{k-j}\frac{Z-k-N+1+j}{Z-k}\end{array}$$

The second summation does not depend on *w*; thus the optimal policy is given by the minimization of:

$$F{{\prime\prime}}=-N\delta \mathop{\sum }\limits_{j=0}^{N-1}\frac{(\begin{array}{c}k-1\\ j\end{array})(\begin{array}{c}Z-k\\ N-1-j\end{array})}{(\begin{array}{c}Z-1\\ N-1\end{array})}(w[\frac{a}{j+1}-\frac{b}{N-j}\frac{k}{k-j}\frac{Z-k-N+1+j}{Z-k}])$$

**Figure 4:**
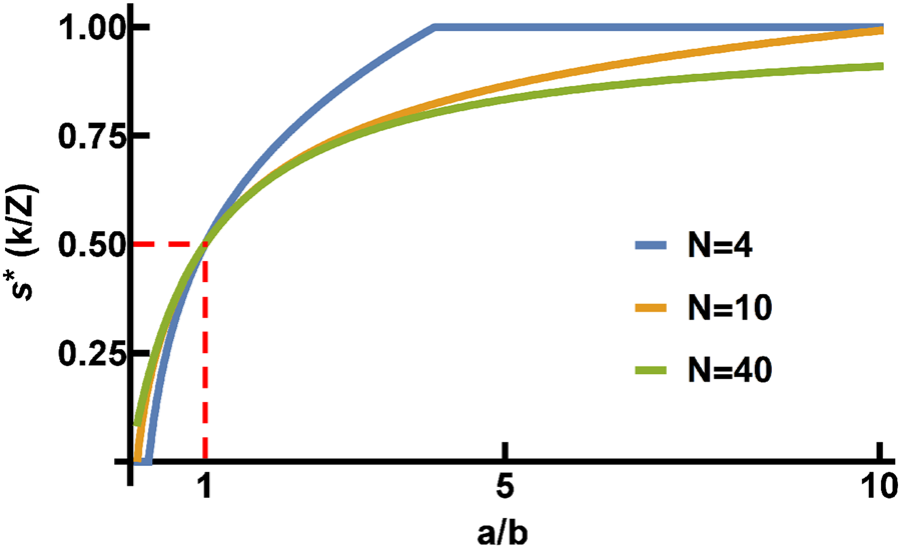
Optimal switching point s* as a function of the ratio *a*/*b*, for different values of *N* (see Methods).

Since *N* and *δ* are always positive, the whole expression can be divided by *N*δ without changing the optimization problem. Moreover, by multiplying the expression by (−1), it can finally be shown that minimizing *f*_D_ − *f*_C_ is equivalent to maximizing the following expression:

$$w\mathop{\sum }\limits_{j=0}^{N-1}\frac{(\begin{array}{c}k-1\\ j\end{array})(\begin{array}{c}Z-k\\ N-1-j\end{array})}{(\begin{array}{c}Z-1\\ N-1\end{array})}([\frac{a}{j+1}-\frac{b}{N-j}\frac{k}{k-j}\frac{Z-k-N+1+j}{Z-k}])$$

where *j* represents the number of ***C***s in a group of size *N*, sampled without replacement from a population of size *Z* containing *k Cs*. Now, let us consider that the optimal switching point s* depends on *k*. Since this sum decreases as *k* increases, containing only one root, the solution to this optimization problem corresponds to having *w* set to 1 (pure Reward) for positive values of the sum, suddenly switching to w = 0 (pure Punishment) once the sum becomes negative. The optimal switching point s* depends on the ratio $\frac{a}{b}$, group size *N* and population size *Z*. The effect of population size (*Z*) and group size (*N*) on s* is limited, while the impact of the efficiency of reward (*a*) and punishment (*b*) is illustrated in Fig. 4. For $\frac{a}{b}=1$ the switching point is s* = 0.5 (see Fig. 4). Interestingly, we note that, also in the **CRD**, s* is not impacted by the group success threshold (*M*) or the risk associated with losing the retained endowment when collective success is not attained (*r*). This is the case as we assume that the decision to punish or reward is independent on *M* or *r*. Notwithstanding, the model that we present can, in the future, be tuned to test more sophisticated incentive tools, such as rewarding or punishing depending on (*i)* how far group contributions remained from (or surpassed) the minima to achieve group success or (*ii)* how soft/strict is the dilemma at stake, given the likelihood of losing everything when collective success is not accomplished.
